# Appendicitis

**Published:** 1896-02

**Authors:** S. W. S. Toms

**Affiliations:** Bellport, L I., N. Y.; Fellow of the New York Academy of Medicine


### APPENDICITIS.

### Illustrative Cases with Histories of their Course.—Results of Medical and Surgical Treatment Compared.

By S. W. S TOMS, Ph. G., M. D., Bellport, L I., N. Y.
Fellow of the New York Academy of Medicine.

THE following cases are reported because of the lessons they convey in connection with this most interesting disease as to the course they pursued both before and after treatment, together with the relation of the conditions we may reasonably expect resulting from acute attacks, primary or recurrent.

Especially are they of interest just now, because of the general acceptance of sound surgical principles in their management, when the reports of so many series of cases of successful operations *during the intervals of attacks* by eminent American surgeons in various parts of the country which have appeared in current medical literature within a year from such men as Senn, McGuire, Murphy and others.

Three years ago there were good men in all parts of the United States who contended against the prompt operative treatment in acute attacks, and it has been stated the treatment of appendicitis has given rise to more controversy[1] in medical annals than any other disease known. Happily, it has passed the transition stage and many opponents of operation, and many others who were "on the fence" have accepted the logic of experience and now renounce the old medical fad of ultra-conservatism for just and good reasons—no doubt of a personal nature. Time, too, coupled with the widespread reports of the public press, has had its effect on the lay mind, and patients or their friends rarely now cling to the hope of trusting to Nature, but prefer to take the chances of recovery by operation. Furthermore, it is clearly evident, cases that have providentially passed through an acute attack readily accept the proposition to have an operation before another one occurs, preferring the slighter risk to that of the greater with all its attending and lingering incomplete convalescence.

My cases are not reported in chronological order, but they all occurred during my first year's practice in a rather small community.

Case I.—*Peritonitis, (?) so-called. No operation. Treatment by opiates. Death.*—February 21, 1893, I saw J. T. O'C., a bright, intelligent lad, aged 6 years, of a good family and personal history; had been ailing for two days previous with cramps, at intervals, which were increasing in severity. The night before he frequently awakened and cried out with pain. I learned that previous to the onset he had ingested a quantity of dried sweet corn, and had received a fall against a platform, striking his abdomen. He did not complain much at the time, but some ten hours afterward he was seized with abdominal pain. A dose of a patent medicine containing principally aloes was administered by the mother, as his bowels had been constipated. The bowels moved slightly in half an hour, probably not from the action of the medicine. He vomited several times before the movement and the cramps disturbed him frequently. These symptoms continued for two days, when I found the abdomen flat, fairly relaxed, excepting when in pain ; at those times he would flex his thighs on the abdomen and curl himself up, crying out from the pain. His bowels had not moved. (I was not told of the circumstance of the fall, or that he had eaten the dried corn until some days after). The pain he located in the epigastrium. There was no tenderness complained of upon palpation and no tumor existed in any region that could be made out. Deep pressure in the right iliac region was slightly painful, although the same was complained of when made in the opposite side. Pulse, 90 ; temperature, 100° F. He had been unable to retain scarcely any nourishment since he was first taken. His tongue was coated with a whitish fur and was dry ; skin dry and countenance at times anxious. Small and frequent doses of calomel were given at intervals during the night and a dose of oil in the morning ; this latter was rejected. The child passed a better night than the preceding one. In the morning I gave a high-up enema of Epsom salts, turpentine, glycerine and soapsuds, through a soft rubber rectal tube in the genu-pectoral posture ; only a few hard, dark-colored scybala passed, mixed with corn shells, also a little blood-stained mucus. I gave small doses of antispasmodics, combined with minute doses of morphia, and at night 30 c.c. of castor oil in egg emulsion. He had small quantities of milk and lime-water, most of which was retained.

The oil increased the cramps and vomiting soon became troublesome again. I was at this time informed of the injury and about the dried corn eaten three days before. On the following morning, finding the patient with a tense abdomen and constantly flexed legs, I requested counsel. Peritonitis was the diagnosis made and the plan of treatment suggested was opiates, liquid nourishment and stimulation. The urine was abundant, of the "beery" appearance; the temperature ranged about the same with some acceleration of pulse and respirations. The following day patient's abdomen was tympanitic and his condition still more unfavorable. A second consultation with another practitioner was requested by the family. The diagnosis and treatment were concurred in and Tully's powder given to maintain the respirations at about seven to ten per minute. Hot stupes were also applied to the abdomen.

On the fifth day he was taking 0.01 of morphia every hour. The pulse continued to rise to 140 and became weaker and more irregular, but the temperature fell to about 99.5°. Marked icterus developed on the sixth day and he required to be catheterised for forty-eight hours before death. Death occurred on the ninth day and was due to unmistakable general septic peritonitis, undoubtedly from rupture of abscess or perforation of the vermiform appendix. No autopsy was permitted.

From the history as above related it might be inferred by many who have this only for a guide that this case might have been one of intestinal obstruction, or volvulus. However, the course of symptoms indicated septic peritonitis, which was rapid after undoubted peritoneal infection.

CASE II.—*Gangrenous appendicitis with perforation. Septic peritonitis. Operation. Death.*—At midnight, June 20, 1893, I saw C. P. P., aged 50, American, capitalist, married, who was suffering from cramps. He had lost one brother after an operation for appendicitis. Patient always had good health, excepting a weak stomach; although a high liver, he was not a drinking man. From adolescence he had had frequent attacks of "cramps" within the abdomen. In the mornings he frequently suffered from nausea and vomiting.

Two days previous to the above date he fell violently to the ground, while playing tennis, inflicting a slight sprain in one ankle; but the fall apparently greatly prostrated him and he suffered from shock for several hours after, the symptoms being out of all proportion to the accident. He vomited and was unable to eat any supper. He was taken with violent abdominal pain, paroxysmal in character, the next evening, twenty-four hours before I saw him. He informed me he was attended by a physician, who administered morphia hypodermatically and the skin was reddened by the effects of a large sinapism applied to the whole abdomen.

He was around town attending to business the day following the fall and preceding the attack, and felt no inconvenience, except for the painful and tender ankle. In the evening he returned to his summer cottage here and I was called at midnight as stated. He was apparently suffering acutely with diffused abdominal pain; deep pressure over the right inguinal region elicited great tenderness. I diagnosticated a probable appendicitis, administered an anodyne and left him for the night. There was slight elevation of temperature; pulse, 90; bowels constipated.

I was called again about six A. M., as he was again having pain. I gave an enema, bringing away hard masses of scybala. Administered .50 of calomel to be followed in two hours by a teaspoonful of sulphate of magnesia every half hour in concentration, until free evacuations were induced. Three movements were produced before one o'clock.

Although the paroxysms were mitigated the vomiting persisted at intervals. Morphia was given hypodermatically to allay this annoying symptom, as nothing whatever partaken of was retained. During the afternoon two more movements of the bowels followed, which consisted of clear, uncolored mucus, perfectly free from fecal matter. The abdomen was tenser and the right side hard and tender. No tumor could be made out. Patient had several rigors; pulse, 110; temperature, 96° F. Rectal examination by finger elicited pain on the right side. His family physician, Dr. Francis W. Murray, of New York, was wired to come at once, prepared to operate for appendicitis. At that time some abdominal distension was perceptible. All nourishment was rejected by the stomach during the day, the pulse became more rapid and thready, and the countenance more anxious. Morphia was given by needle to put the patient at ease and restrain peristalsis. Ice was kept applied to the abdomen. Drs. McBurney and Murray arrived in the evening. After consultation the patient was etherised at eleven o'clock. The operation lasted about forty minutes, the incision made in the usual way. When the peritoneum was incised, pus and serum spurted upward several inches. Pus was free in the abdominal cavity. The appendix was in a state of gangrene with three perforations. No limiting adhesions were found and the most intense peritonitis with paralysis of the bowels, allowing of great distension, pervaded the entire abdominal contents. The abdomen was thoroughly cleaned out from all septic fluid as was possible and large quantities of hot normal saline solution were poured in among the intestines, which were freed from lymph and washed thoroughly, drainage provided and the wound allowed to remain open.

The patient stood the operation well and recovered consciousness within an hour after being placed in bed. He died of general septic peritonitis fifty-two hours after operation, despite all efforts to overcome intestinal paralysis.

This case was one of those that appear, from the results of so rapid gangrene, fatal from the onset; although, had he been operated on within a few hours of the first attack of colic, peritonitis, possibly, might have been averted and a chance of recovery possible.

CASE III.—*Appendicitis. Recovery (?) without operation. Recurrent symptoms following.*—August 9, 1893, J. B. S., aged 39, single, hotel proprietor, American; family history good and with no history of specific disease. He was suddenly attacked with intestinal cramps located mostly in the right side, which was especially sensitive to palpation. Percussion elicited hyperresonance, although by deep pressure a well-defined tumor could be felt in the cecal region and the entire colon distended with feces could be distinctly mapped out. Constipation had existed for several days, with irregularity and unsatisfactory evacuations for some time previous. Patient said he never had suffered from a similar attack. His tongue was coated and dry; temperature, 101° F.; pulse, 100; abdomen, soft; pain was quite sharp and vomiting occurred. A full dose of calomel, followed by salines, failed to move the bowels until a high-up enema of glycerine, salts solution and turpentine brought away large quantities of hard fecal matter; free purgation followed. As the symptoms ameliorated, the patient was kept in bed upon liquid diet and morphia administered. All signs of the acute stage passed off, excepting extreme tenderness in the right iliac region, which remained for over a month. Nine months after he informed me pain or an uneasy feeling follows exertion. For this reason recovery is certainly incomplete.

CASE IV.—*Appendicitis. Recovery (?) without operation. Spontaneous evacuation of abscess per rectum.*—February 8, 1894, W. S., aged 34, German; peddler; married. Family history indifferent. Previous illness: an attack of rheumatism five years ago; other than that has always been healthy. On above date patient was exposed to a severe chilling. Early the next morning he was taken with severe cramps. Was not constipated up to onset of attack, but obstinately so after its inception. Vomiting supervened, when one attack was soon followed by another paroxysm. The pain was referred to the abdomen, generally, but more especially to the right inguinal region. A tumor was palpable, the size of a fetal head at the sixth month; hard and tender, giving a dull percussion note. Only moderate rigidity of the abdominal muscles on the right side existed. The man lay with his legs flexed. The skin was hot and dry. Temperature, 101° F.; pulse, 120. One gramme of calomel was given, followed in three hours by 5.0 of sulphate of magnesia in concentration every hour for several doses, to be assisted after the fourth dose, if necessary, by a high-up enema in the knee-chest position, of the composition given in the other cases. Three such injections of about half an hour intervals were given before an evacuation was effected, when a large amount of scybala was expelled. A fourth injection was given when soft fecal matter followed. A short interval elapsed when desire to go to stool again came on. At this movement about a teacupful of bloody pus was evacuated. Following this several watery passages occurred during the day and succeeding night. After this all symptoms subsided and convalescence was uninterrupted. This was a primary attack. A year after, this man informed me he was not well. His bowels are irregular, his side is weak and tender, he is unable to do hard work because of the fear of bringing on another attack and he is constantly troubled with hemorrhoids.

CASE V.—*Appendicitis. Recovery (?) without operation.*—December 7, 1893, C. C., aged 36, American; carpenter; married. Excellent family and personal history. Patient says he has never had any illness excepting two or three attacks of "inflammation of the bowels"; and, when a youth, had a number of attacks of "wind colic" and diarrhea. He constantly suffers from constipation and such was his condition for several days before I was called to attend him at day-break one morning, for an attack of cramps, which had set in the night before after supper. An hour or two after the onset of pain he vomited, which recurred several times during the night. Domestic remedies and hot applications to abdomen failed to afford relief. When I saw him he was suffering great agony, was much concerned and depressed by the sudden seizure; there was some shock, temperature was subnormal, pulse small and rapid. I administered a simple gastric sedative, containing a small amount of morphia, and directed he should have the powder (containing 1 gm. calomel) when he became somewhat more tranquil. The calomel was to the followed by salines, as is my custom, until free catharsis ensued. I saw him again in six hours and found he had not had an evacuation. He had become easier, the vomiting had ceased and he retained all the medicines. I gave a high-up enema which was very successful soon afterward; free evacuations followed. These measures relieved as they had in the previous cases and the patient reacted quickly. In this case no tumor was present, the abdomen was flat and very tense and tender, pain was diffuse, but the point of maximum tenderness was over cecum and McBurney's point. He stated he afterward had "crampy" suggestions in his right side for weeks; and six months, subsequently, I found him wearing a porous plaster over the old seat of trouble as a prevention of recurrence—a very suggestive sign even to him that he had *not recovered.*

CASE VI.—*Perforative appendicitis and diffused peritonitis. Operation. Recovery. With comments.*—A. D. H., aged 24, single, American; student, with a family history of intestinal disorders, was taken ill with cramps and vomiting on the night of July 2, 1893. I was informed by the patient that he had a maternal uncle die of appendicitis, also a brother, aged 7, of the same disease following an operation. A sister, aged 11, then living, had several attacks in a mild form.[1] His father and mother are living and well—the latter was at this time convalescing from an operation for tumor of the breast. At the bedside I learned that this present attack was a recurrence of many previous similar ones, but more violent in its onset. Apparently it had been precipitated by the ingestion of some articles of food taken the previous evening for dinner which invariably disagreed with him, and which enjoined upon him great care in the regulation of his diet. There

1. NOTE.—Since the above was written this sister has died of "anorexia nervosa." The autopsy revealed a perfectly healthy appendix—despite the fact several attacks from which she suffered were diagnosticated appendicitis. She would have been operated upon but for the conservatism of the family physician, although he did not dissent from the diagnosis.

existed a previous history of constipation preceding this attack—of irregularity of the bowels and unsatisfactory movements. The patient was not robust in appearance and had a dull complexion, a loaded skin. Eight years previous, while in the south of France, he had an attack of "inflammation of the bowels" which confined him to his bed for three weeks and from which he made a slow and tedious convalescence. He was treated by opiates and poultices. That was the last and most severe attack previous to the present one. He informed me he was suddenly awakened about midnight with severe cramps in his abdomen, shortly followed by vomiting. These symptoms were paroxysmal and persistent throughout the night. The pain, although diffuse, was mostly referred to the region of the umbilicus. I saw him about 9.30 A. M. (July 3d), and found him suffering profoundly from shock, with an anxious countenance, a pinched face and blanched lips. A cold, clammy perspiration exuded from the extremities; pulse small and compressible, but not rapid, and temperature slightly subnormal. The abdomen was flat and the right rectus muscle tense; tenderness was seemingly as diffused as the pain. Deep pressure over the entire abdomen was not especially painful. A rectal examination was made later in the day, eliciting pain and tenderness at the brim of the pelvis on the right side. The psoas muscle on the right side was in a state of spasm. The diffuse abdominal tenderness did not become acute until it became localised in the right iliac region. The subsidence of general tenderness into an acute localised area occurred on the second day of the disease; subsequently recurring over the hypogastrium, denoting very conclusively the progress of peritonitis, from infection after rupture or perforation.

At no time was there tenderness above the umbilicus or over the liver. At the onset the pain was acute and general; later it became dull and more localised. According to Bryant,[2] the character of pain experienced denoted the involvement, primarily, of intraperitoneal tissues, directly affecting the splanchnic nerves, giving rise to the generalised character of pain and tenderness. Subsequently, the abdominal sympathetic nerves became paretic and then only the dull pain of connective tissue implacation was manifest. This symptom answered most closely the description given by Fitz:[3] "Sudden severe abdominal pain is the most constant, first decided symptom of perforating inflammation of the appendix." There was no pain in the testicle of the right side, and only in the bladder and rectum after peritonitis had involved those structures on the fourth day. Tumor could never be made out by palpation or percussion.

The urine was abundant and of high color. The course of disease, its treatment and termination were as follows: To relieve intestinal peristalsis, eight dosimetric granules of 0.0000625 of hyoscyamine with one of 0.0005 of strychnia were administered every half hour and 0.60

of bismuth subnitrate with 1 c. c. of chloranodyne as gastric sedatives were at once administered. I called again in about three hours and found the paroxysms somewhat ameliorated, the patient having had some sleep and had reacted from shock. In the evening the patient was easier, but with a rise of temperature to 100° F. and pulse marking 82 per minute. He had been practically unable to retain any nourishment, vomiting frequently during the day. The pain was less severe. The bowels had not moved. An enema was given with negative results.

To induce rest for the night, 0.01 of morphia sulphate was given hypodermatically, on which he rested reasonably well. On the morning of the 4th, I found him somewhat refreshed; he had vomited once or twice, but had retained some peptonised milk; temperature was 100.5°; pulse, 100. Pain decidedly relieved and the tenderness more localised in the right iliac region. Calomel in 0.012 doses was given every half hour for ten doses, to be followed by 5.00 of sulphate of magnesia in concentration every half hour, for three or four doses. The third dose was rejected by the stomach. The bowels had not moved and a soap and water enema did not induce a good movement. A few hours after, a high-up Epsom salts, glycerine and turpentine enema was given with a rectal tube with satisfactory results and the expulsion of considerable gas and hard dark-colored scybala. The patient was not allowed at any time to rise from the dorsal decubitus. Early in the afternoon I acquainted the friends with the seriousness of the case and requested counsel. Dr. Wm. H. Draper, of New York, was summoned and confirmed my diagnosis and agreed as to the necessity of early operation. Dr. Chas. McBurney was wired for, but he was out of town and did not arrive until 5 o'clock the following day.

During that day the patient had partaken of small quantities of peptonised milk, most of which were retained. In the evening the pulse was 125 and temperature 102°. He had not had any chills, but had had some slight rigors. A hypodermic injection of morphia sulphate 0.015 was given at 9 P. M. and some tablets of the same size left to be given by mouth if necessary before morning. One or two were given, but the patient had a poor night, was very restless and vomited several times.

On July 5th, the morning temperature was 102°; pulse, 135. The paroxysms of pain had ceased, the abdomen was still flat, the tenderness was marked on the right side and extended over the hypogastrium. By the aid of 0.015 doses of cocaine muriate, given fifteen minutes before taking the peptonised milk, he retained considerable nourishment and passed a more comfortable day. An ice bag was retained over the right inguinal region from the time the diagnosis was made, which added much to the patient's comfort. Two or three hypodermic injections of morphia 0.01 were given during the day to maintain quietness and splint the bowels to favor formation of adhesions. Pain was complained of in urination. In the evening the condition of the patient had not materially changed, although the pulse was less rapid, being 100, and temperature 100°. On the arrival of Drs. McBurney and Francis W. Murray, at 6 o'clock P. M., July 5th, all preparations necessary having been completed, the patient was etherised and the operation commenced forty minutes after the arrival of the surgeons. A four-inch incision was made in the line of the linea semilunaris, and upon incising the peritoneum, thick greenish pus boiled out through the opening to the amount of about 60 c.c.

This was carefully wiped away and the abscess cavity explored digitally, which revealed an ulcerated appendix nearly severed in two. Two fecal concretions, sharp and with flat surfaces, the size of 1.25 c.m. by .75 c.m., had escaped and were floating in the pus. The abscess cavity was not walled off by any adhesions.

After evacuation of the thick fetid pus a quantity of sanious purulent fluid followed and the pelvis was carefully wiped out with dry sterile sponges. The appendix separated when raised to pass a ligature around the base. After cauterizing the stump and covering it with peritoneum it was dropped back into place and the peritoneal cavity thoroughly irrigated with a hot saline solution. The pelvic cavity contained several ounces of effusion; and lymph adhered to coils of intestines that were brought into view. Drainage was provided for by numerous strips of iodoform gauze, ramifying in all directions among the intestines and into the pelvis. A glass drainage-tube also was carried into the pelvic cavity. The external wound was left open with the drainage gauze protruding and the application of a large amount of antiseptic absorbent dressings completed the operation.

It became necessary to remove the outer layers of dressings on the following day, as the amount of drainage had completely saturated the entire dressings.

The patient recovered from the anesthetic and became perfectly conscious within an hour after being placed in bed. He only vomited once after the operation and did not require any morphia whatever after the first forty-eight hours. He slept and rested well most of the first night and voluntarily voided considerable urine at 5 A. M. A nutritive enema was given every four hours for two days; and after the full effects of the ether had passed off, small quantities of milk and vichy were allowed by mouth every hour or two. The patient scarcely complained of any pain or discomfort, rested quietly and improvement was rapid.

He was removed to his home on August 17th, and the wound entirely and firmly closed by August 27th. By September 1st he was practically well.

This gratifying termination—wholly unexpected at the time of operation by Dr. McBurney, and shared by Dr. Murray and myself—goes far to confirm the latter-day methods of surgical technique.

A case like this should dispel all arguments against the necessity of operation or hesitancy in doing it *early*. Had this been instituted three days earlier, no doubt it would have saved the patient much time in his convalescence.

We are hearing daily from a very large number of intelligent physicians arguments which, to those who employ them, at least seem reasonable. Yet, these same gentlemen will admit in a general way that pus pent up should be evacuated wherever it may be located. Should this not be a thousand times more so when the general peritoneal cavity—that great absorbing sac !—is in danger of being compromised ? Go still further : Is it not much better to lessen the chances of death by appreciating the condition in its earlier stages and applying the first principles of prophylaxis ?

We often hear at the consultation, " How can these terminations —of pus, perforation, ulceration, rupture, gangrene, and the like—be recognised to justify radical measures at the outset ? " It may be that the patient has had previous attacks and recovered (?). He may have absence of temperature and no tumor or fluctuation possible to determine—in fact, he may have a subnormal temperature and the pulse may give no indication of serious trouble or involvement of vital structures. But these are not arguments of logic ; they are born of inexperience and of misconception of the actualities which clinical cases demonstrate daily—often fatally.

Bacteriological investigations[4] have clearly demonstrated the fact that an appendix once infected is forever afterward a diseased organ until the lymphoid coat is destroyed.[5] If a recovery (?), so-called, has followed an attack, the lymphoid tissue retains the latent infection. If such be so—and why should a doubt exist on such evidence of the bacteriologist, verified daily by clinical examples,—what advantage is there in procrastination of operation in the face of existing acute symptoms ? The danger today of exploring the peritoneal cavity in such cases where grave doubt might exist, is almost nil when properly and carefully executed. It is an imperative duty to give a patient every chance of life and the highest ideal is the prevention of a recurrence of a disease. In the light of past experience it is manifestly more dangerous to defer and " trust to nature " than to explore where doubt exists. The appendix vermiformis is a useless organ in our race, and is a constant source of danger to life in a large proportion of mankind. It can be removed without danger of compromising life and its removal does not encompass after-effects to anything like the risks of allowing it to remain diseased after attacks of appendicitis have occurred.

If one or more attacks have occurred, the appendix is more extensively diseased and its structures weakened; hence, it becomes manifestly more imperative in our early recognition of the disease to operate at once.

Temperature, fluctuation and tumor when not present do not constitute pathognomonic signs against the possibility of encysted abscess, ulceration leading on to perforation or gangrene with general septic infection.

There are few diseases that are more insidious in their onset at times; or more deceiving and difficult of recognition in respect to the actual condition, as it may exist wherein every moment adds to the danger of perforation or rupture of weakened tissues from previous attacks, which may have left old cicatrised ulcers or adhesions—the consequent contractions interfering with the circulation in a deformed appendix.

Perforation or slow suppurative processes from secondary infection of tubercle bacilli may develop, covering a considerable interval, and giving rise to scarcely any general disturbances until a fatal rupture of an abscess into the peritoneal sac occurs; or, a septic phlebitis of the mesenteric veins from thrombi or emboli; or, abscess of the liver; or, septic emboli swept into the lungs with abscess, gangrene or empyema, are possible consequences and may develop with lethal rapidity, defeating and defying the best efforts to save a patient's life; nor is a patient with a diseased appendix a safe risk for insurance.

The saddest commentary on conservative surgery (so-called) is for the patient to die. Perhaps, the next is an imperfect recovery and delayed convalescence when both are avoidable in the vast majority of these cases. It seems possible for a patient to recover from a severe general peritonitis, arising from a ruptured pus tube of gonorrheal origin, and occasionally, too, from a puerperal peritonitis, but never from an acute general septicemia from pyogenic infection with pus from a suppurative or gangrenous appendicitis.

It seems strongly inconsistent in those surgeons who admit these principles and are governed by them in general surgical work, for them to oppose their application in this disease.

Year by year, the so-called "expectant plan" of treating disease is happily yielding to means born of better judgment, which research conducted in the wider fields of investigation, coupled with the aid of science and experience, is furnishing.

It is coming to light from the personal experiences of those who have "recovered" (?) from previous attacks of appendicitis, without operation, that the conservative measures which caused pus to become absorbed or encapsulated, (if they lived long enough,) inevitably have a recurrence and generally in an aggravated form as in other suppurative processes. As Morris has truly said, "Catarrhal appendicitis is a misnomer;" and as there is no pathological proof to the contrary, but great clinical proof that such does not exist, confirmed by the revelations of the microscope, it is better to give the patient the benefit of the doubt than speculate on a special form of catarrh never seen.

---

1. Murphy, *Medical News*, January 5, 1895.
2. Paper, New York State Med. Ass'n, Feb. 7, 1894.
3. *American Journal of the Medical Sciences*, October, 1886.
4. Morris, Robt. T., paper read at Pan-American Medical Congress, 1893.
   Hadenpyl, E., *New York Medical Journal*, December 30, 1893.
   Fowler, Geo. R., *New York Medical Journal*, October 14, 1893.
5. Senn, N., *Journal of American Medical Association* for March 24, 1894.

